# Low expression of KIF20A suppresses cell proliferation, promotes chemosensitivity and is associated with better prognosis in HCC

**DOI:** 10.18632/aging.203494

**Published:** 2021-09-06

**Authors:** Chuanxing Wu, Xiaosheng Qi, Zhengjun Qiu, Guilong Deng, Lin Zhong

**Affiliations:** 1 Department of General Surgery, Shanghai General Hospital Affiliated to Shanghai Jiaotong University, Shanghai, China

**Keywords:** HCC, hub genes, prognosis, KIF20A, proliferation

## Abstract

This study analysed the microarray datasets from Gene Expression Omnibus (GEO) database, and aimed to identify novel potential hub genes associated with the progression of HCC via bioinformatics analysis and experimental validation. The common differentially expressed genes (DEGs) from five GEO datasets were screened using GEO2R tool. The expression and survival analysis of hub genes in HCC were performed using Gene Expression Profiling Interactive Analysis, UALCAN and Kaplan-Meier plotter tools. *In vitro* functional assays were used to determine the caspase-3, -9, cell proliferation and chemo-sensitivity of HCC cells. A total of 177 common DEGs were identified between normal liver and HCC tissues among these datasets. Functional enrichment and PPI network analysis identified 22 hub genes from the common DEGs. The mRNA expression of 22 hub genes was all significantly up-regulated in HCC tissues compared to that in normal liver tissues. Further survival analysis showed that 10 hub genes predicted poor prognosis of patients with HCC. More importantly, the *in vitro* functional studies demonstrated that KIF20A knockdown suppressed the HCC cell proliferation and promoted the chemosensitivity of HCC cells to cisplatin and sorafenib. In conclusion, the present study identified a total of 177 common DEGs among 5 GEO microarray datasets and found that 10 hub genes could predict the poor prognosis of patients with HCC using the comprehensive bioinformatics analysis. Furthermore, KIF20A silence suppressed cell proliferation and enhanced chemosensitivity in HCC cells. Further studies may be required to determine the mechanistic role of these hub genes in HCC progression.

## INTRODUCTION

Liver cancer represents a type of frequent human malignancy responsible for the mortality in both men and women worldwide [1]. In China, every year nearly 0.5 million people are diagnosed with liver cancer, and around 0.3 million people die from this disease [2]. Hepatocellular carcinoma (HCC) is the main type of liver cancer. Various factors including nonalcoholic steatohepatitis, alcoholism, viral infection and smoking are shown to contribute to the development of HCC [3]. The 5-year overall survival (OS) of these patients is less than 30% and varies among different populations [3]. Up to date, the current treatment strategies for HCC are quite limited [3]. Moreover, the high heterogeneity in HCC as well as the complex risk factors make the prognosis prediction very difficult. Therefore, it is of paramount importance to further explore possible mechanisms underlying the pathophysiology of HCC.

Recently, omics studies have been conducted to explore possible mechanisms underlying the HCC pathophysiology [4, 5]. The great progress in high-throughput technologies have enabled the scientists to reconstruct the regulatory signaling pathways involved in cancer biology [4, 5]. Using microarray screening techniques, differentially expressed gene (DEG) profiles between normal and cancerous tissues have been identified in HCC studies. Studies have performed the high throughput microarray analysis in different HCC stages, which could be key to identify potential biomarkers of for HCC prognosis [6, 7]. Zhang et al., performed bioinformatics analysis and found that 9 weighted genes participated in HCC pathophysiology [8, 9]. Zhang et al., performed analysis using The Cancer Genome Atlas (TCGA) RNA sequencing data and found that transketolase and olfactomedin 2 could serve as novel prognostic biomarker for HCC patients [10]. Xiao et al., performed the bioinformatics analysis and experimental validation showing that upregulation of centromere protein M promotes hepatocarcinogenesis through multiple mechanisms [11]. However, due to the large amount of the existing microarray datasets, sufficient mining of these datasets has been a great challenging in the HCC studies. Thus, more efforts should be invested to further explore these microarray datasets, in order to decipher the complex molecular mechanisms underlying HCC pathophysiology.

This explored the Gene Expression Omnibus (GEO) microarray datasets related to HCC progression. In this study, a final of 5 microarray datasets were included the analysis. The common DEGs compared between HCC tissues and normal tissues among all these datasets were identified. These common DEGs were subjected to enrichment analysis to reveal their potential actions in HCC progression. Furthermore, the hub genes were derived from the protein-protein interaction (PPI) network constructed from common DEGs. Expression profiles and prognostic potentials of the hub genes in HCC were further explored using series of online tools including Gene Expression Profiling Interactive Analysis (GEPIA) [12], UALCAN [13] and Kaplan-Meier (KM) plotter [14] databases, respectively. Finally, Kinesin Family Member 20A (KIF20A), one of the hub genes, was further subjected to the experimental validating studies, so as to confirm the role of KIF20A in the pathophysiology of HCC.

## RESULTS

### Screening of common DEGs among GSE84598, GSE87630, GSE101685, GSE101728 and GSE121248 microarray datasets

**Figure 1:**
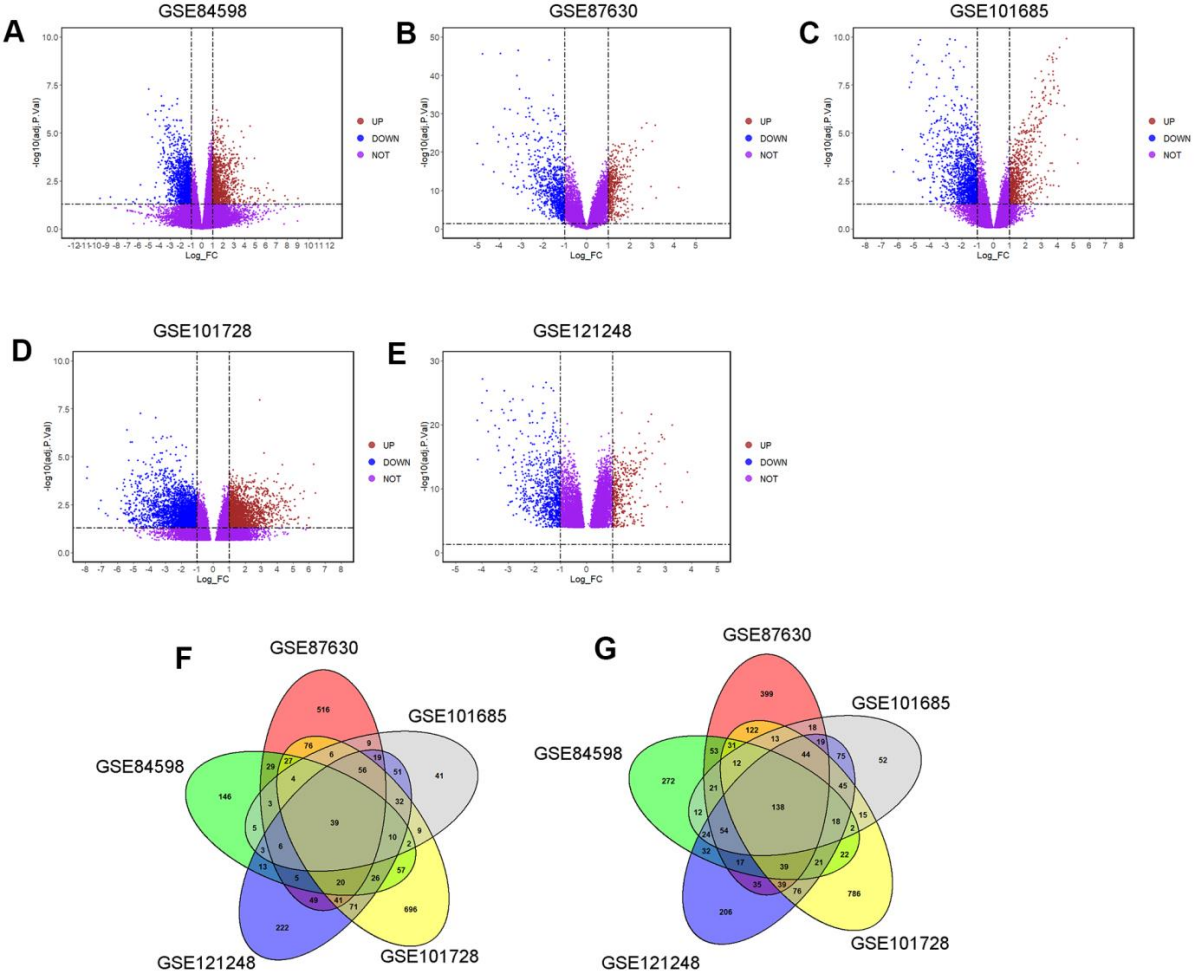
**Identification of common DEGs among GSE84598, GSE87630, GSE101685, GSE101728 and GSE121248 microarray datasets.** The DEGs in datasets: (**A**) GSE84598, (**B**) GSE87630, (**C**) GSE101685, (**D**) GSE101728 and (**E**) GSE121248 were visualized by the volcano plots. UP = up-regulated DEGs; DOWN= down-regulated DEGs; NOT = Not-significantly DEGs. (**F**) Venn diagram of the up-regulated common DEGs among the five datasets. A total of 39 up-regulated common DEGs was screened. (**G**) Venn diagram of the down-regulated common DEGs among the five datasets. A total of 138 down-regulated common DEGs was screened.

The DEGs in different microarray datasets were analyzed using GEO2R tool. In GSE84598, 1163 DEGs (up-regulation: 395 and down-regulation: 768) were screened between normal liver and HCC tissues (Figure 1A); in GSE87630, 1959 DEGs (up-regulation: 905 and down-regulation: 1054) were screened between normal liver and HCC tissues (Figure 1B); in GSE101685, 829 DEGs (up-regulation: 284 and down-regulation: 545) were screened between normal liver and HCC tissues (Figure 1C); in GSE101728, 2596 DEGs (up-regulation: 1172 up-regulated and down-regulation: 1424) were screened between normal liver and HCC tissues (Figure 1D); in GSE121248, 1545 DEGs (up-regulation: 663 and down-regulation: 882) were screened between normal liver and HCC tissues (Figure 1E). As shown in Figure 1F, 1G, 39 up-regulated and 138 down-regulated common DEGs among the five datasets were detected.

### Enrichment analysis of common DEGs

Common DEGs were first subjected to Gene Ontology (GO) enrichment analysis. Common DEGs were significantly enriched in “carboxylic acid catabolic process”, “small molecule metabolic process”, “organic acid catabolic process”, “detoxification of copper ion” and so on of the biological process category (Supplementary Figure 1A); in the “pore complex”, “membrane attack complex”, “extracellular vesicle", ”extracellular space” and so on of the cellular component category (Supplementary Figure 1B); in the “monooxygenase activity”, “oxidoreductase activity”, “reduction of molecular oxygen”, “heme binding” and so on of molecular function category (Supplementary Figure 1D). Common DEGs were significantly enriched in KEGG pathways including “tryptophan metabolism”, “prion diseases”, “mineral absorption”, “metabolic pathways”, “complement and coagulation cascades” and “caffeine metabolism” (Supplementary Figure 1D).

### PPI network construction and hub genes screening using MCODE

**Figure 2:**
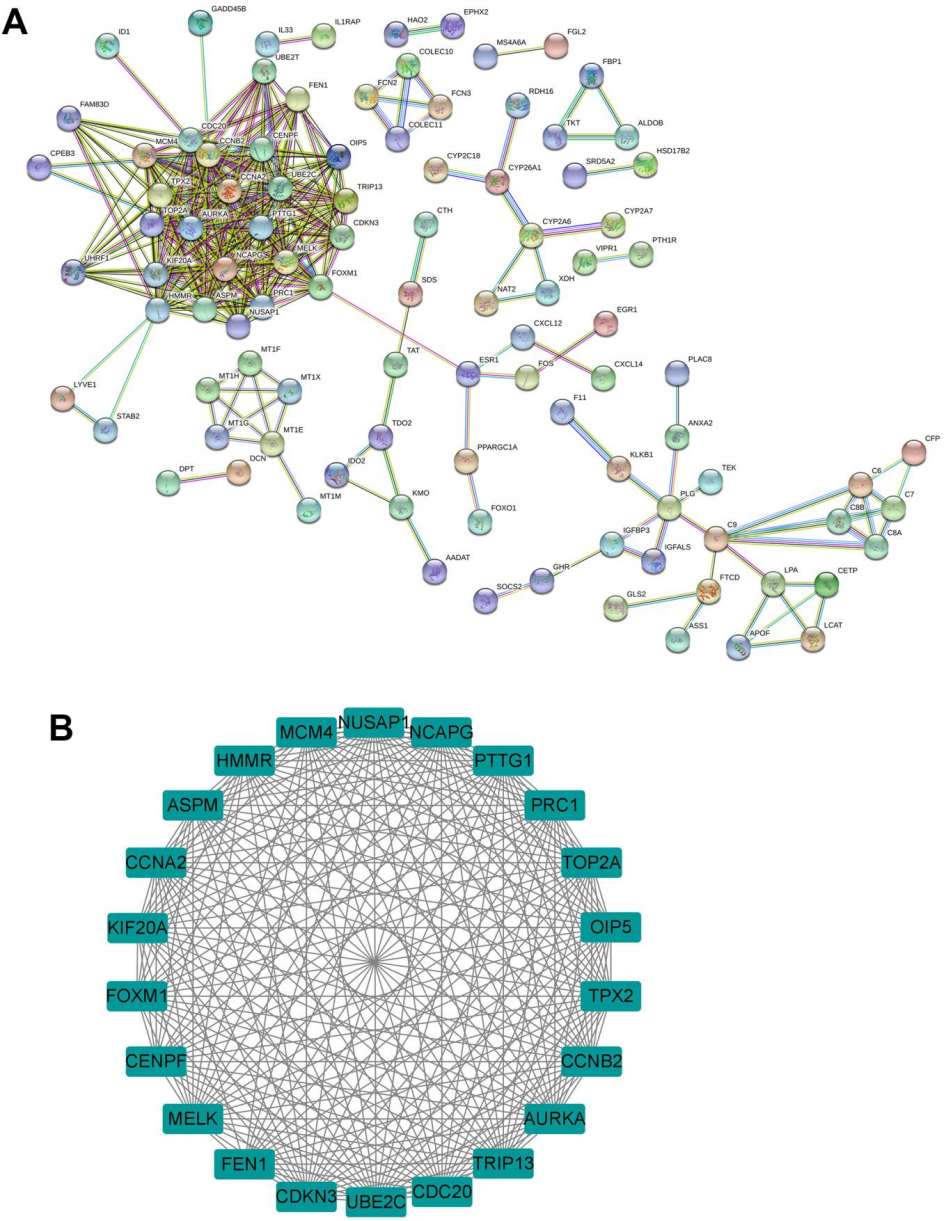
**Identification of hub genes from common DEGs.** (**A**) PPI network of the common DEGs was constructed by the STRING database. (**B**) The sub-module of PPI network as identified by the MCODE tool in Cytoscape.

PPI network of the common DEGs was constructed using the Search Tool for the Retrieval of Interacting Genes (STRING) database. Constructed PPI network contains 175 nodes and 346 edges with an average node degree of 3.95 (PP enrichment P-value < 0.001; Figure 2A). Furthermore, the interacted genes were imported into the Cytoscape software, and the MCODE was used for the module analysis. As shown in Figure 2B, one sub-module with MCODE score greater than 5.0 was identified, and this module has a MCODE score of 21.333 and contains 22 nodes and 224 edges (Figure 2B). As such, the 22 genes in this module were selected for the expression and survival analysis.

### Hub gene expression analysis

**Figure 3:**
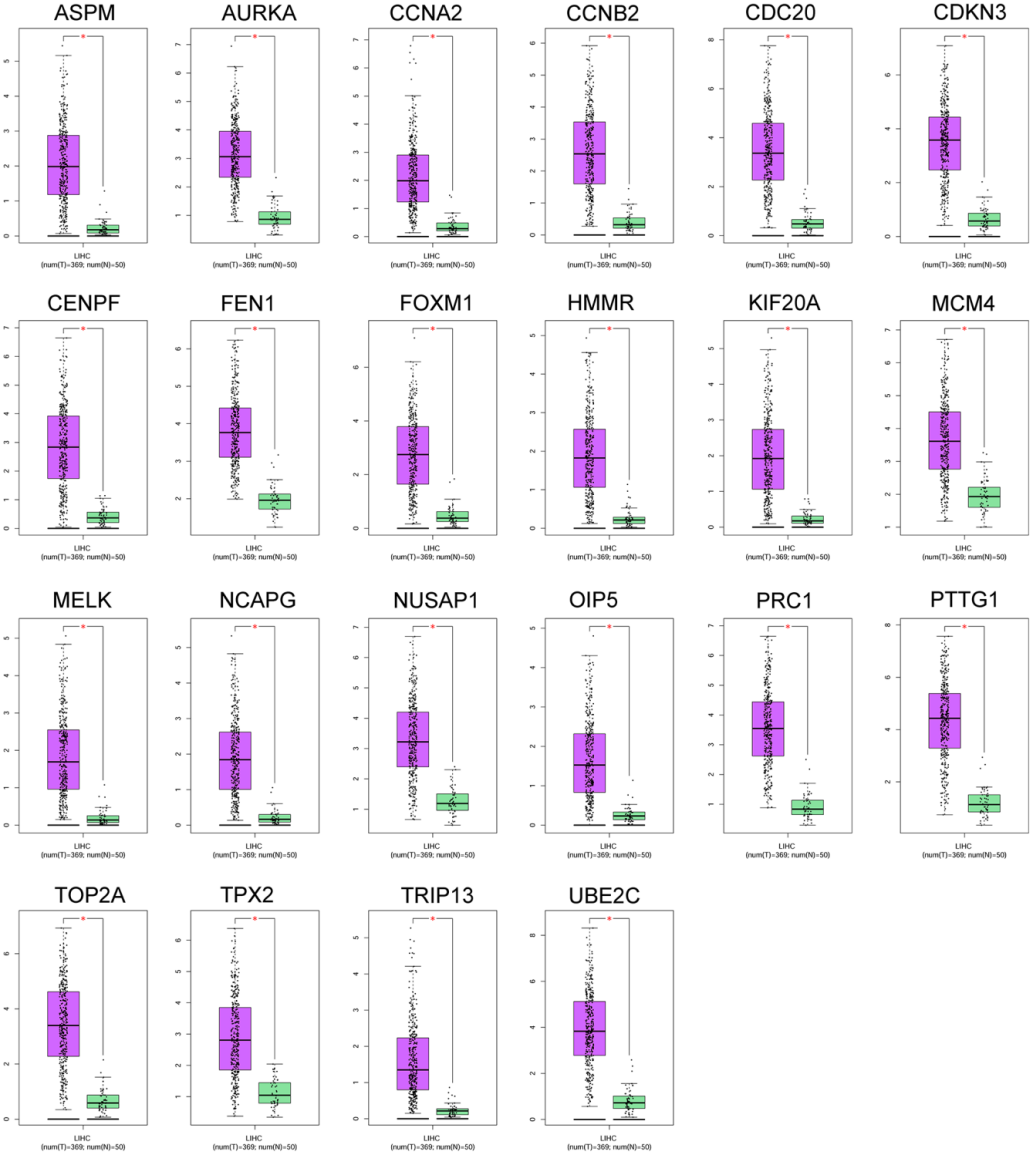
**The mRNA expression of 22 hub genes between normal liver tissues and HCC tissues was analyzed by the GEPIA tool.** The genes including ASPM, AURKA, CCNA2, CCBN2, CDC20, CDKN3, CENPF, FEN1, FOXM1, HMMR, KIF20A, MCM4, MELK, NCAPG, NUSAP1, OIP5, PRC1, PTTG1, TOP2A, TPX2, TRIP13 and UBE2C were analyzed. N = normal liver tissue group (n = 50); T = HCC tissues group (n = 369); *P<0.05.

Expression levels of 22 hub genes were analyzed using GEPIA and UALCAN databases. The 22 hub genes (ASPM, AURKA, CCNA2, CCBN2, CDC20, CDKN3, CENPF, FEN1, FOXM1, HMMR, KIF20A, MCM4, MELK, NCAPG, NUSAP1, OIP5, PRC1, PTTG1, TOP2A, TPX2, TRIP13 and UBE2C) were up-regulated n HCC tissues comparing normal liver tissues (Figure 3). Consistently, mRNA expression of 22 hub genes was analyzed by using UALCAN databases, and the heatmap showed that these genes were up-regulated in HCC tissues (Supplementary Figure 2).

### Survival analysis of hub genes

**Figure 4:**
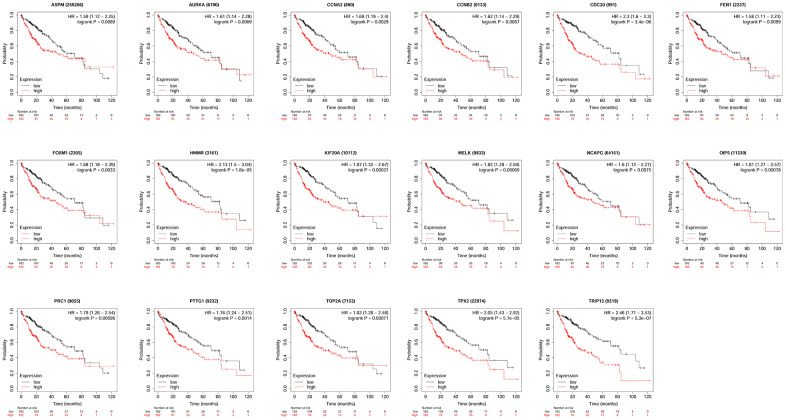
**The correlation between the expression of 17 hub genes (APSM, AURKA, CCNA2, CCNB2, CDC20, FEN1, FOXM1, HMMR, KIF20A, MELK, NCAPG, OIP5, PRC1, PTTG1, TOP2A, TPX2 and TRIP13) and the overall survival of patients with HCC was analyzed by KM plotter.** A total of 364 patients with HCC (low expression = 182 and high expression = 182) were included in the analysis.

**Figure 5:**
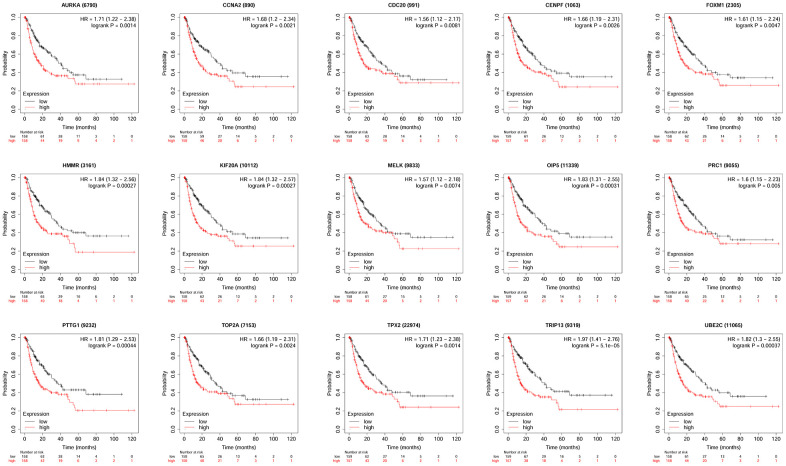
**The correlation between the expression of 15 hub genes (AURKA, CCNA2, CDC20, CENPF, FOXM1, HMMR, KIF20A, MELK, OIP5, PRC1, PTTG1, TOP2A, TPX2, TRIP13 and UBE2C) and the disease-free survival of patients with HCC was analyzed by KM plotter.** A total of 316 patients with HCC (low expression = 158 and high expression = 158) were included in the analysis.

**Figure 6:**
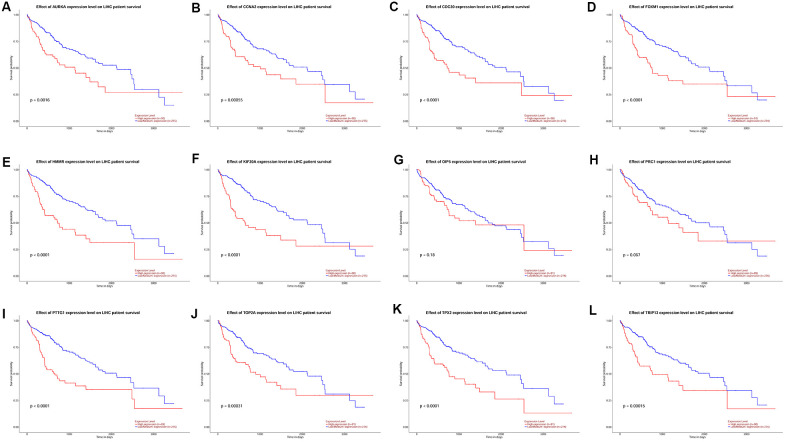
The correlation between the expression of 12 hub genes AURKA (**A**), CCNA2 (**B**), CDC20 (**C**), FOXM1 (**D**), HMMR (**E**), KIF20A (**F**), OIP5 (**G**), PRC1 (**H**), PTTG1 (**I**), TOP2A (**J**), TPX2 (**K**) and TRIP13 (**L**) and the disease-free survival of patients with HCC was analyzed by UALCAN database. A total of 365 patients with HCC (low/medium expression = 274 and high expression = 91) were included in the analysis.

KM plotter database was used to perform survival analysis. Among the 22 hub genes, the up-regulated 17 genes (APSM, AURKA, CCNA2, CCNB2, CDC20, FEN1, FOXM1, HMMR, KIF20A, MELK, NCAPG, OIP5, PRC1, PTTG1, TOP2A, TPX2 and TRIP13) were correlated with the shorter OS of patients with HCC (Figure 4); while the other 5 hub genes were not correlated with the OS of patients with HCC. Furthermore, the 17 hub genes were subjected to disease-free survival (DFS) analysis using KM plotter, and the up-regulated 15 hub genes (AURKA, CCNA2, CDC20, CENPF, FOXM1, HMMR, KIF20A, MELK, OIP5, PRC1, PTTG1, TOP2A, TPX2, TRIP13 and UBE2C) were correlated with shorter DFS of HCC patients (Figure 5). Based on the above results, 12 hub genes (AURKA, CCNA2, CDC20, FOXM1, HMMR, KIF20A, OIP5, PRC1, PTTG1, TOP2A, TPX2 and TRIP13) predicting both poor overall survival and DFS of patients with HCC were further confirmed using UALCAN databases. As shown in Figure 6, the up-regulation of AURKA (Figure 6A), CCNA2 (Figure 6B), CDC20 (Figure 6C), FOXM1 (Figure 6D), HMMR (Figure 6E), KIF20A (Figure 6F), PTTG1 (Figure 6I), TOP2A (Figure 6J), TPX2 (Figure 6K) and TRIP13 (Figure 6L) was significantly correlated with shorter OS; while OIP5 (Figure 6G) and PRC1 (Figure 6H) were not correlated with the OS. Thus, 10 hub genes including AURKA, CCNA2, CDC20, FOXM1, HMMR, KIF20A, PTTG1, TOP2A, TPX2 and TRIP13 may predict poor prognosis of the patient with HCC.

### Effects of KIF20A knockdown on the cell proliferation and caspase-3 and -9 activities of HCC cells

**Figure 7:**
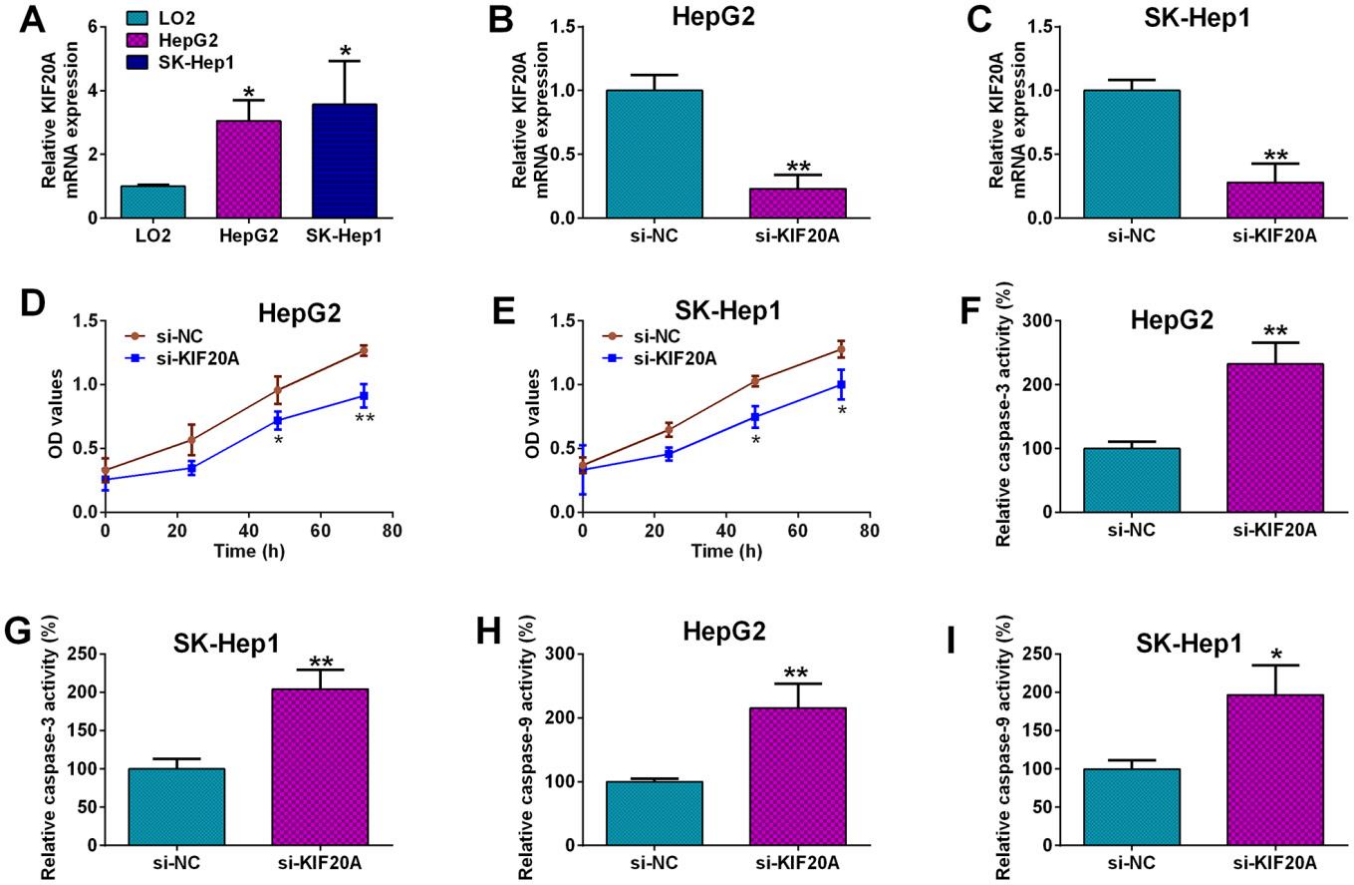
**Effects of KIF20A knockdown on the HCC cell proliferation and capsase-3 and -9 activities.** (**A**) qRT-PCR analysis of KIF20A mRNA expression in LO2, HepG2 and SK-Hep1 cells. (**B**, **C**) qRT-PCR analysis of KIF20A mRNA expression level in HepG2 (**B**) and SK-Hep1 cells (**C**) transfected with si-NC or si-KIF20A. (**D**, **E**) MTS assay analysis of cell proliferation of HepG2 (**D**) and SK-Hep1 cells (**E**) transfected with si-NC or si-KIF20A. (**F**, **G**) Capsase-3 activity analysis of caspase-3 activities of HepG2 (**F**) and SK-Hep1 cells (**G**) transfected with si-NC or si-KIF20A. (**H**, **I**) Capsase-9 activity analysis of caspase-9 activities of HepG2 (**H**) and SK-Hep1 cells (**I**) transfected with si-NC or si-KIF20A.

Among these genes, KIF20A has not been fully explored, thus, role of KIF20A in the HCC pathophysiology was further explored. KIF20A expression was remarkably up-regulated in the HCC cell lines i.e. HepG2 and SK-Hep1 when compared to normal liver cell line i.e. LO2 (Figure 7A). Furthermore, the *in vitro* functions of KIF20A in HCC cells were evaluated by the loss-of-function studies. The knockdown of KIF20A in both HepG2 and SK-Hep1 cells was achieved by transfecting these cells with KIF20A; the transfection of KIF20A siRNA significantly decreased the mRNA expression level of KIF20A in HepG2 and SK-Hep1 cells (Figure 7B, 7C). The MTS proliferation assay revealed that silence of KIF20A significantly attenuated the proliferative capacities of both HepG2 and SK-Hep1 cells as compared to the si-NC group (Figure 7D, 7E). Caspase-3 and -9 activity kits were used to measure caspase-3 and -9 activities in HCC cells, respectively. As shown in Figure 7F and 7G, KIF20A knockdown significantly increased the caspase-3 activities in both HepG2 and SK-Hep1 cells (Figure 7F, 7G); and consistent findings for caspase-9 were also detected in the HCC cells with KIF20A siRNA transfection (Figure 7H, 7I).

### Effects of KIF20A knockdown on the chemosensitivity of HCC cells

**Figure 8:**
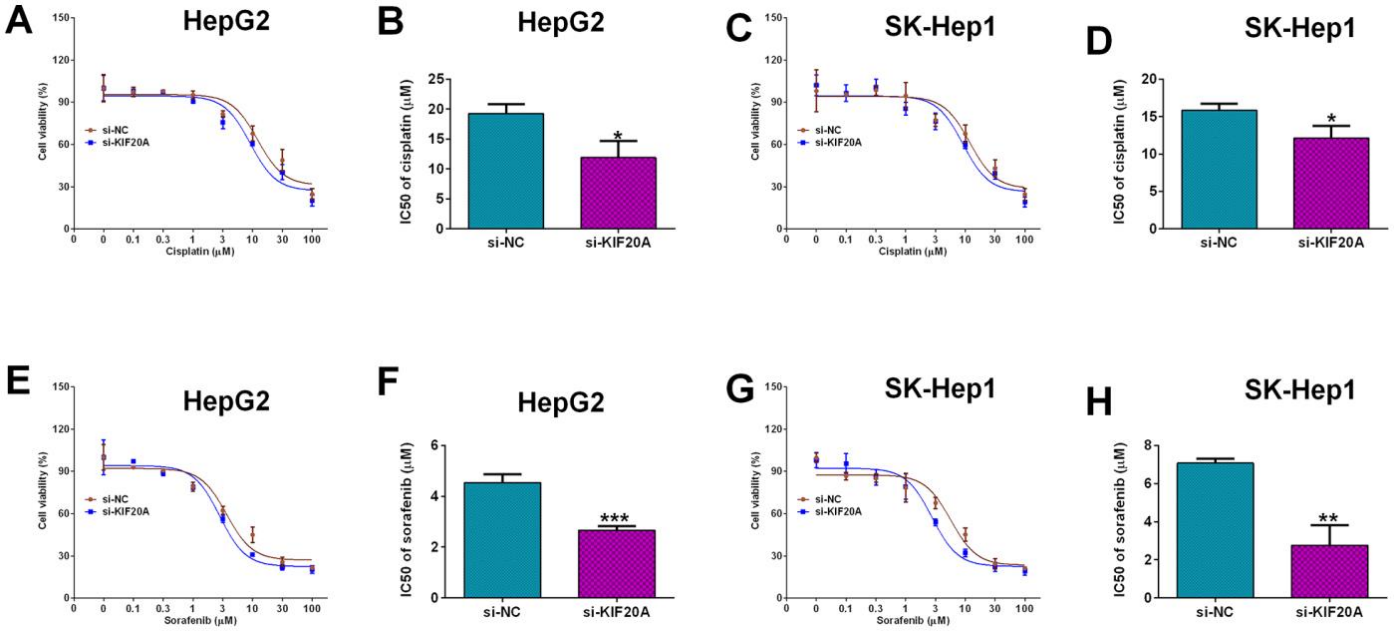
**Effects of KIF20A knockdown on the chemo-sensitivity of HCC cells.** (**A**) Cell viability of si-NC or si-KIF20A-transfected HepG2 cells after treatment with different concentrations of cisplatin. (**B**) IC50 values of cisplatin in HepG2 cells transfected with si-NC or si-KIF20A. (**C**) Cell viability of si-NC or si-KIF20A-transfected SK-Hep1 cells after treatment with different concentrations of cisplatin. (**D**) IC50 values of cisplatin in SK-Hep1 cells transfected with si-NC or si-KIF20A. (**E**) Cell viability of si-NC or si-KIF20A-transfected HepG2 cells after treatment with different concentrations of sorafenib. (**F**) IC50 values of sorafenib in HepG2 cells transfected with si-NC or si-KIF20A. (**G**) Cell viability of si-NC or si-KIF20A-transfected SK-Hep1 cells after treatment with different concentrations of sorafenib. (**H**) IC50 values of sorafenib in SK-Hep1 cells transfected with si-NC or si-KIF20A. N = 3; *P<0.05, **P<0.01 and ***P<0.001.

Furthermore, the effects of KIF20A knockdown on the cisplatin-resistance and sorafenib-resistance were analyzed in HepG2 and SK-Hep1 cells. Knockdown of KIF20A significantly increased the chemo-sensitivity to cisplatin in HepG2 cells when compared to si-NC group with the si-KIF20A group exhibiting decreased IC50 values of cisplatin (Figure 8A, 8B); consistent findings were also found in SK-Hep1 cells (Figure 8C, 8D). More importantly, knockdown of KIF20A significantly decreased the IC50 values of sorafenib in both HepG2 (Figure 8E, 8F) and SK-Hep1 cells (Figure 8G, 8H) when compared to si-NC group, suggesting the KIF20A increased the chemo-sensitivity to sorafenib in HCC cell lines.

## DISCUSSION

HCC represents a type of frequent human malignancy, and patients with advanced HCC had poor prognosis, though great improvements have been made in the HCC treatment [3]. Recently, the high-throughput technologies using microarray datasets have significantly promoted the discovery of novel targets associated with HCC pathophysiology [5]. Yet, more efforts should be investigated to reveal potential mechanisms underlying HCC progression. This study collected five microarray datasets associated with HCC, and identified a total of 177 common DEGs between normal liver and HCC tissues among these datasets. Further functional enrichment and constructed PPI network screened 22 hub genes from the common DEGs. The 22 hub genes were significantly up-regulated in HCC tissues. The survival analysis showed that 10 hub genes including AURKA, CCNA2, CDC20, FOXM1, HMMR, KIF20A, PTTG1, TOP2A, TPX2 and TRIP13 were correlated with poor prognosis of HCC patients. More importantly, functional studies demonstrated that KIF20A knockdown suppressed HCC cell proliferation and increased the HCC cell chemosensitivity to cisplatin and sorafenib. Collectively, these results indicated that these screened hub genes could potential serve as the key biomarkers for HCC.

This study revealed that 10 hub genes were associated with shorter overall survival and DFS of patients with HCC. Among these hub genes, AURKA, CCNA2, CDC20, TOP2A and TPX2 were associated with the cell division and cell cycle transition based on the functional enrichment analysis. Overexpression of AURKA and TPX2 enhanced HCC progression [15–17], and up-regulation of these genes predicted worse prognosis of patients with HCC [18], which were consistent with our findings. CCNA2 is a member of cyclin family and participates in modulating cell cycle; knockdown of CCNA2 attenuated the HCC tumor growth [19], and CCNA2 activation predicted poor prognosis of HCC [20]. CDC20 interacts with the anaphase-promoting complex/cyclosome in cell cycle and involves in tumor progression. Studies have found that high CDC20 expression might contribute to HCC progression [21], and predicted worse prognosis in HCC patients [22], which was consistent with our findings. TOP2A is a key mediator in regulating chromosome segregation, DNA topological structure, and cell cycle progression. TOP2A was found be to overexpressed in HCC tissues [23], and TOP2A overexpression was correlated with early age onset of HCC, shorter HCC patients survival and chemoresistance [24], which is consistent with our findings that TOP2A predicted poor prognosis of HCC. In terms of FOXM1 and TRIP13, they have been well documented for their oncogenic roles in HCC, and FOXM1 and TRIP13 high expression was associated with the poor prognosis of HCC patients [25, 26]. HMMR is highly expressed in various solid tumors and is involved in tumorigenesis [27–29]. Recently, a study demonstrated that HCC patients with HCC with high increased HMMR level had worse prognosis [30], which is consistent with our findings, suggesting that HMMR is likely to serve as a potential biomarker for HCC prognosis.

KIF20A belongs to the KIF superfamily, and aberrant expression of KIF20A was associated with chromosome instability in HCC [9, 31]. Studies have shown that in G2 phase of cell cycle, KIF20A is accumulated in the nucleus, which results in enhanced pathologic hepatocyte proliferation [31]. Studies have showed that KIF20A high expression was linked to poor survival of HCC patients [32–34], which is consistent with our findings. Our *in vitro* functional results revealed that KIF20A silence attenuated the proliferation and increased capsase-3 and -9 activities of HCC cells, consistent with studies showing that KIF20A promoted cell proliferation in pancreatic [35], ovarian [36], bladder [37] and lung cancer [38]; caspase-3 and -9 are two key mediators in the apoptotic signaling pathways, and our results may imply that KIF20A knockdown repressed HCC cell proliferation cells via enhancing activities of cell apoptotic signaling pathway. Actions of KIF20A in the chemo-sensitivity of cancer cells have not been reported. In the CRC, the IC50 values of 5-FU and oxaliplatin were elevated in cells with KIF20A overexpression and decreased with KIF20A silence [39]. In ovarian cancer, KIF20A overexpression conferred epithelial ovarian cancer cells resistance to cisplatin [36]. Consistently, our results showed that knockdown of KIF20A enhanced HCC cell chemosensitivity to cisplatin and sorafenib. Collectively, our results suggested that high expression of KIF02A may confer chemoresistance in HCC, which may lead to poor prognosis of HCC patients.

In this study, *in vitro* functional studies of KIF20A were still limited in the preliminary stage, and further studies may explore down-stream targets of KIF20A in the HCC cells, which may advance our understanding into the role of KIF20A in the HCC development. In addition, *in vivo* studies by investigating the effects of KIF20A on HCC progression are warranted in the future studies. Moreover, other hub gene expect KIF20A are still worthy of further investigation, in order to further decipher HCC pathophysiology.

In conclusion, this study identified 177 common DEGs among 5 GEO microarray datasets, and found that 10 hub genes (AURKA, CCNA2, CDC20, FOXM1, HMMR, KIF20A, PTTG1, TOP2A, TPX2 and TRIP13) could predict the poor prognosis of HCC patients using comprehensive bioinformatics analysis. Our studies for the first time demonstrated that KIF20A silence suppressed cell proliferation and potentiated chemosensitivity to cisplatin and sorafenib in HCC cells. Further studies may be undertaken to decipher the mechanistic role of these hub genes in HCC progression.

## MATERIALS AND METHODS

### Microarray datasets

Five microarray datasets (GSE84598, GSE87630, GSE101685, GSE101728 and GSE121248) analyzed the present study were downloaded from GEO database. In the GSE84598 dataset, the GPL10558 platform was used, and 22 normal liver tissues and 22 HCC tissues were included for analysis. In the GSE87630 dataset, the GPL6947 platform was used, and 30 normal liver tissues and 64 HCC tissues were included for analysis. In the GSE101685 dataset, the GPL570 platform was used, and 8 normal liver tissues and 24 HCC tissues were included for analysis. In the GSE101728 dataset, the GPL21047 platform was used, and 7 normal liver tissues and 7 HCC tissues were included for analysis. In the GSE121248 dataset, the GPL570 platform was used, and 37 normal tissues and 70 HCC tissues were included for analysis.

### Screening of the DEGs

DEGs between normal liver tissues and HCC tissues in the collected datasets were analyzed using the GEO2R tool (https://www.ncbi.nlm.nih.gov/geo/geo2r/), which is an online tool for extracting DEGs. The significant DEGs were screened using the following criteria: up-regulated DEGs have an adjusted *P*-value < 0.05 and log2 fold change (logFC) > 1; down-regulated DEGs have an adjusted *P*-value < 0.05 and logFC < -1. The common DEGs among different datasets were presented by the Venn diagrams.

### Functional enrichment analysis

GO and Kyoto Encyclopedia of Genes and Genomes (KEGG) pathway enrichment analysis of the common DEGs was analysed using the g:Profiler tool [40]. GO terms including biological processes, cellular component and molecular function (P < 0.05 was considered significant enrichment). KEGG is a database resource for revealing high-level functions and effects of the biological pathways. The KEGG pathways with a p value < 0.05 were considered significant enrichment.

### PPI and the module analysis

PPI network was constructed using the STRING database [41], which provides uniquely comprehensive coverage of, and ease of access to experimental and predicted interaction information. A combined score of 0.7 was set as the confidential threshold. Furthermore, the module analysis was performed using the MCODE plugin embedded in the Cytoscape software. The parameters for module analysis using MCODE were as follow: degree cutoff = 2, node score cutoff = 0.2, k-core = 2, and max depth = 100.

### Expression and survival analysis of hub genes

The mRNA expression of hub genes was analyzed using the GEPIA and UALCAN databases. The correlation between expression levels of hub genes and the overall survival (OS) of patients with HCC was analyzed using the KM plotter and UALCAN analysis; the correlation between expression levels of hub genes and the DFS of patients with HCC was analyzed using KM plotter. GEPIA is a web-based tool established for customized investigation of genomic functionalities based on the resources provided by TCGA and the genotype-tissue expression (GTEx) projects; UALCAN database is a portal for facilitating tumor gene expression and survival analyses; KM plotter is a web tool containing Gene expression data and relapse-free and overall survival information derived from the GEO (Affymetrix microarrays only), RNA-sequencing databases, which integrates gene expression and clinical data simultaneously via a PostgreSQL server.

### Cell lines and cell culture

The normal liver cell line (LO2) and the HCC cell lines (HepG2 and SK-Hep1) were obtained from the Chinese Type Culture Collection of the Chinese Academy of Sciences (Shanghai, China). The LO2 cells and SK-Hep1 were cultured in RPMI 1640 medium (Gibco, Waltham, CA, USA) supplemented with 10% heat-inactivated fetal bovine serum (FBS; Gibco); the HepG2 cells were culture in DMEM (Gibco) supplemented with 10% heat-inactivated FBS. All the cells were maintained in a humidified chamber with 5% CO_2_ at 37° C.

### Small interfering RNA (siRNA) transfection and drug treatments in HCC cells

The siRNA designed for silencing KIF20A was purchased from BiboBio (Guangzhou, China). The siRNA sequence for KIF20A was: 5’-GGUGUGAGUAAGCCAGUAU-3’ and the scrambled siRNA was served as the siRNA negative control (si-NC). For the siRNA transfections, cell transfections were done by using the Lipofectamine 3000 reagent (Invitrogen, Carlsbad, CA, USA). The chemicals including cisplatin and sorafenib were purchased from Sigma-Aldrich (St. Louis, MO, USA). For the cisplatin and sorafenib treatment, HCC cells were seeded onto the 96-well plates and were treated with different concentrations of cisplatin or sorafenib for 48 h.

### Quantitative real-time PCR analysis

Total RNA from LO2, HepG2 and SK-Hep1 cells was purified using the TRIzol reagent (Invitrogen). Total RNA was transcribed into cDNA using M-MLV reverse transcriptase (Promega, Madison, USA). The synthetized cDNA was subjected to real-time PCR analysis using the SYBR Green Master Mix kit (Takara, Dalian, China) on an ABI7900 Biosystems (Applied Biosystems, Foster City, CA, USA). The relative mRNA expression of KIF20A was normalized to housekeep gene, GAPDH. The expression level of KIF20A was calculated using the comparative threshold cycle method.

### MTS proliferation assay

Cell proliferation of HCC cells was evaluated using the MTS cell proliferation assay kit (Beyotime). Briefly, after different treatments, HCC cells were seeded in 96-well plate for indicated time durations, and cell proliferation was detected using the MTS proliferation assay kit. Three independent assays were repeated.

### Caspase-3 and caspase-9 activities analysis

The caspase-3 and -9 activities of HCC cells after transfecting with si-NC or si-KIF20A were measured by the commercial caspase-3 and caspase-9 activity assay kits, respectively by following the manufacturer’s protocol. The caspase-3 and caspase-9 assay kits were purchased from Beyotime (Beijing, China).

### Statistical analysis

The *in vitro* data were presented as mean ± standard deviation (SD). The data analysis for the *in vitro* experiments was performed using the GraphPad Prism Software (Version 6.0; GraphPad Software, La Jolla, CA, USA). Significant differences between treatment groups were analysed using unpaired Student’s t-test or one-way analysis of variance followed by Bonferroni’s post-hoc tests. P < 0.05 was considered statistically significant.

### Data availability statement

All the data are available upon reasonable request from the reviewer.

## Supplementary Material

Supplementary Figures
