# Supplementary material for: Low expression of KIF20A suppresses cell proliferation, promotes chemosensitivity and is associated with better prognosis in HCC
Source: Aging (Albany NY). 2021 Sep 6;13(18):22148–63. doi: 10.18632/aging.203494 (PMC8507281; doi:10.18632/aging.203494)
Supplement: Supplementary Figures [file aging-13-203494-s001.pdf]

SUPPLEMENTARY FIGURES

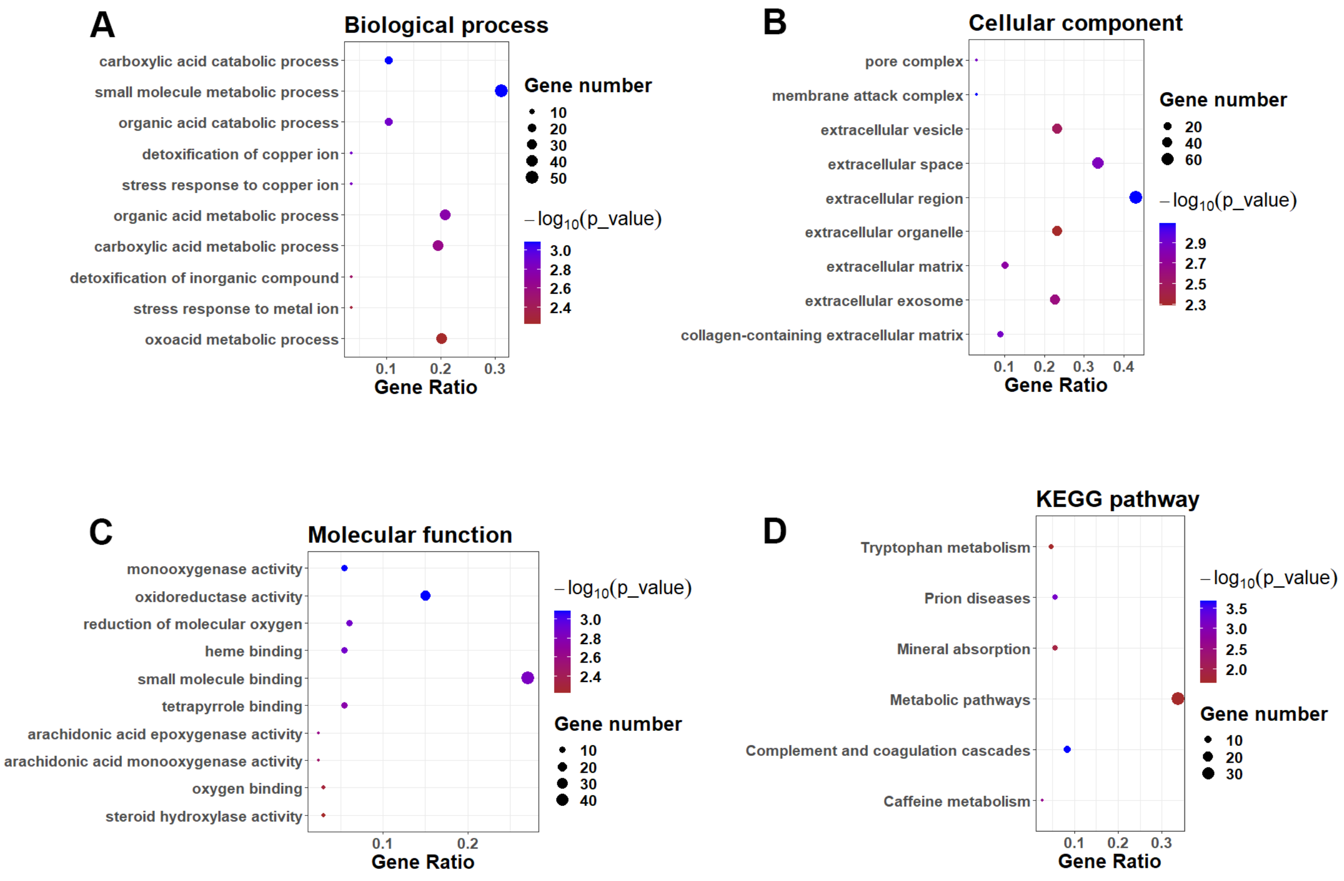

Supplementary Figure 1. Functional enrichment analysis of common DEGs. GO enrichment of common DEGs in (**A**) biological process, (**B**) cellular component and (**C**) molecular function. (**D**) KEGG pathway enrichment analysis of common DEGs.

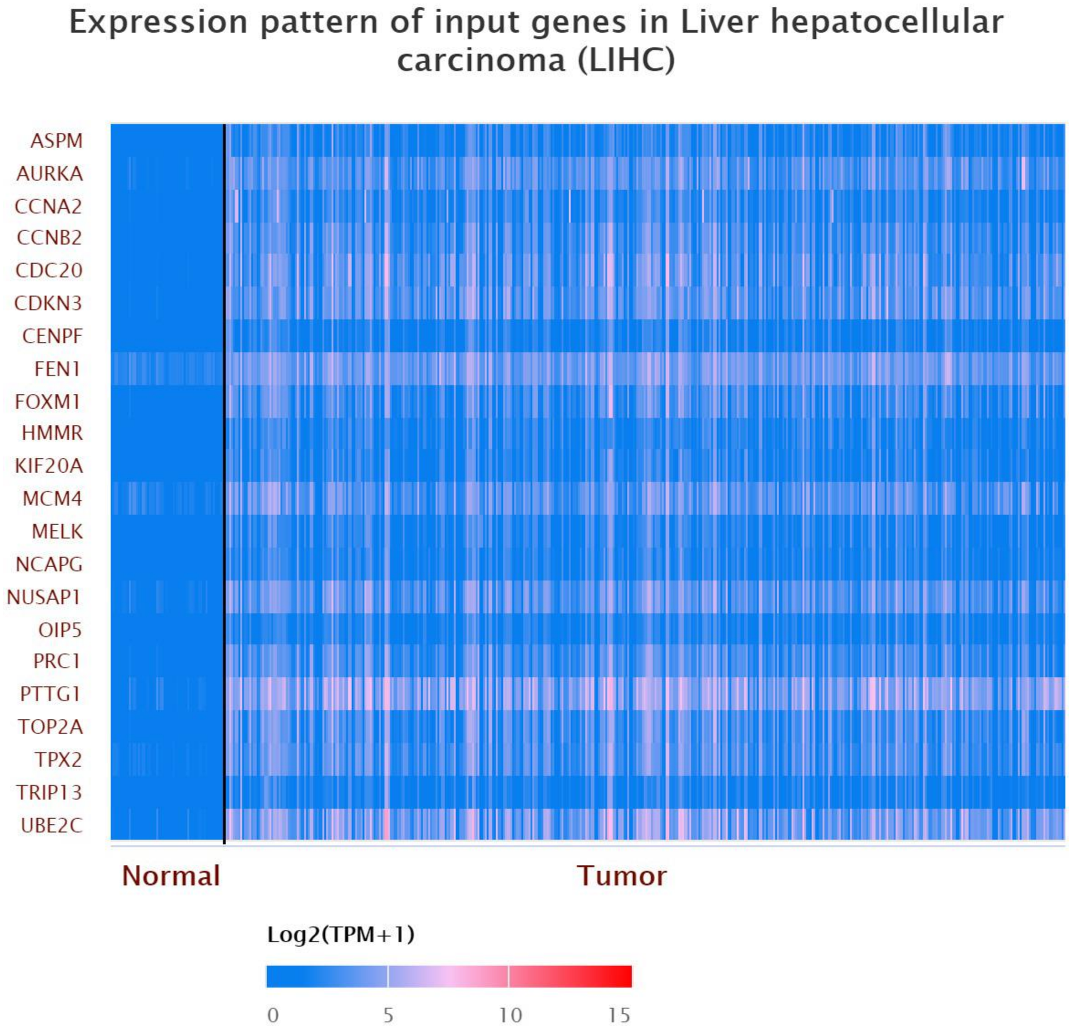

Supplementary Figure 2. Heatmap illustrates the expression pattern of hub genes in normal liver tissues and HCC tissues. TPM = transcripts per million.
